# Quadricuspid Aortic Valve: A Rare and Incidental Finding

**DOI:** 10.7759/cureus.6054

**Published:** 2019-11-02

**Authors:** Mandeep Kaur, Htoo Kyaw, Cesar Ayala-Rodriguez, Sarath Reddy

**Affiliations:** 1 Internal Medicine, Brooklyn Hospital Center, Brooklyn, USA; 2 Cardiology, Brooklyn Hospital Center, Brooklyn, USA; 3 Cardiology, Brooklyn Hospital Center - Mount Sinai Heart, Brooklyn, USA

**Keywords:** quadricuspid aortic valve, pregnancy, aortic regurgitation, transthoracic echocardiography

## Abstract

A quadricuspid aortic valve (QAV) is an exceedingly rare congenital heart anomaly with around 200 cases reported in the literature since the first case was reported in 1862. To the best of our knowledge, there has not been any case of QAV associated with pregnancy. We report a case of a 29-year-old female with new-onset palpitations, diagnosed with QAV and mild aortic regurgitation during pregnancy. The patient presented with new-onset intermittent palpitations at the 37th week of pregnancy. Electrocardiogram (EKG) showed normal sinus rhythm, and transthoracic echocardiography revealed a quadricuspid aortic valve with three equal-sized cusps and one smaller cusp and mild aortic regurgitation without any additional anomalies. QAV morphology falls under the category type b Hurwitz & Roberts classification. She underwent normal vaginal delivery without any peripartum cardiac complications.

In conclusion, QAV is a rare congenital anomaly. It is not uncommon to be associated with aortopathies. The presence of QAV and associated anomalies in pregnancy makes it a high-risk state. Close monitoring, especially during the second and third trimesters, remain of utmost importance. Because of its rarity, the characteristics, natural history, and long-term outcomes of QAV are poorly defined.

## Introduction

The quadricuspid aortic valve (QAV) is one of the rare cardiac conditions and its incidence in the general population ranges from 0.013%-0.043%. [1] We conducted a literature review for the incidence of QAV during pregnancy and discovered that no similar case has been reported thus far. In 1973, Hurwitz and Roberts introduced the classification system in which seven subtypes, named A to G by the relative size of the four cusps, have been classified [2-3]. QAV is often found to be associated with various other congenital anomalies We hereby report a case of a 29-year-old healthy female, discovered to have a quadricuspid aortic valve at 37 weeks of gestation.

## Case presentation

**Figure 1:**
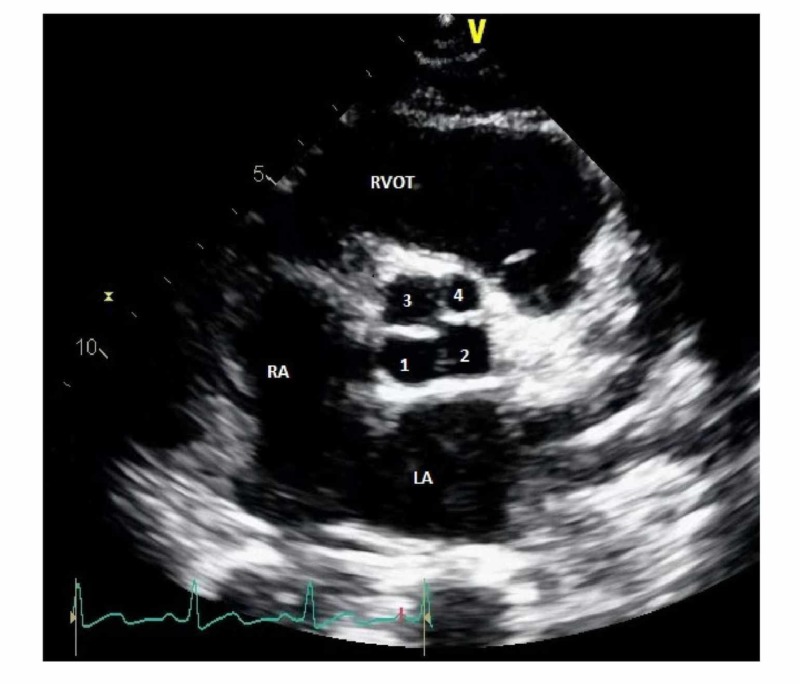
TTE showing the quadricuspid aortic valve Transthoracic echocardiogram (parasternal short axis) showing a quadricuspid aortic valve 1: noncoronary cusp; 2: left coronary cusp; 3: right coronary cusp; 4: accessory cusp; RA: right atrium; LA: left atrium; RVOT: right ventricular outflow tract

**Figure 2:**
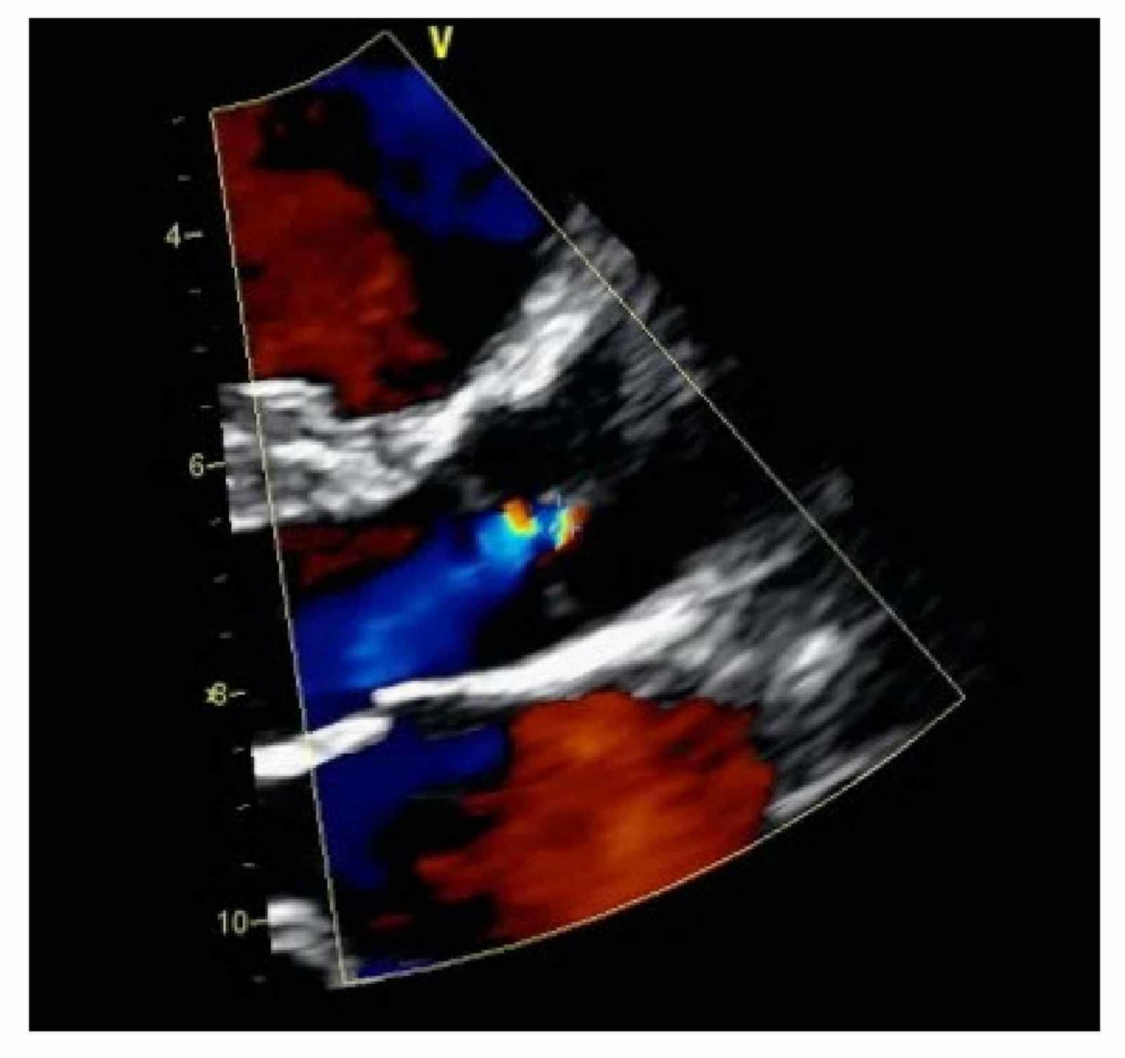
Transthoracic color Doppler echocardiogram showing mild aortic regurgitation

A 29-year-old female in her 37th week of pregnancy was referred to the cardiology clinic for palpitations that started about one to two weeks before presentation. She described the palpitations as intermittent in nature, without any dyspnea, chest pain, or other associated symptoms. Medical history was neither significant for a prior history of medical diseases nor a family history of heart disease. Vital signs were recorded as a heart rate of 80 bpm, blood pressure of 99/61 mmHg, and respiratory rate of 14 per minute. On exam, there was a regular heart rate with normal S1 and S2 without any extra heart sounds. EKG showed normal sinus rhythm. Transthoracic echocardiogram showed normal left and right ventricular size and systolic function, a quadricuspid aortic valve with three equal-sized cusps, and one smaller cusp (Figure 1) and mild aortic regurgitation (Figure 2). No other structural abnormalities were noted. She was advised to follow up at intervals of four to six weeks at the outpatient cardiology clinic for an assessment of volume status and aortic regurgitation. She underwent an uneventful vaginal delivery without any peri- or postpartum complications and was discharged home after three days of hospitalization. Ongoing follow-up with a cardiologist was recommended to monitor the post-partum complications of QAV in the setting of aortic regurgitation.

## Discussion

The first quadricuspid aortic valve was described in 1862 by Balington in an autopsy report [1-5]. It can appear as an isolated defect or in association with other cardiac anomalies, such as anomalous origin and/or coronary course, interatrial and/or interventricular septal defect, patent ductus arteriosus, mitral valve prolapse, Fallot tetralogy, partial atrioventricular canal, subaortic stenosis, non-obstructive hypertrophic cardiomyopathy, and ascending aortic aneurysm [4-5]. QAV has been classified into seven subtypes named A to G by the relative size of the four cusps [6-7].

According to a Mayo clinic study comprising 50 patients, aortic regurgitation (AR) was present in 90%. Of these, 26% had moderate or severe AR at the time of diagnosis. Only four patients (8%) had aortic stenosis, which was mild in all four [8]. Our patient was found to have AR, which was mild in severity. Since our patient had palpitations on presentation, we found out that literature reports cases of the association of QAV with cardiac arrhythmia. A patient was found to have a QAV with paroxysmal supraventricular tachycardia [9]. Moreno R et al. from Spain reported a case of congenital complete atrioventricular block in a 36-year-old-woman who presented with syncope. She was found to have associated QAV. She underwent permanent pacemaker implantation for symptomatic complete heart block [10]. Our patient was found to be in sinus rhythm with no arrhythmias.

We could explain the etiology of palpitations in our case at the 37th week of gestation by the relative hemodynamic changes that can occur in the pregnancy. The total blood volume increases throughout pregnancy. The vasodilation, along with the increase in total blood volume, leads to a volume-overload state. An approximately 50% increase in cardiac output, together with physiologic four-chamber dilatation, occurs [11-12]. As annular aortic dilatation can occur with the increased volume load during pregnancy, the severity of regurgitation may increase. However, in the setting of normal left ventricular systolic function, mild valvular regurgitant lesions are well-tolerated [12].

For symptomatic aortic regurgitation during pregnancy, The American College of Cardiology’s (ACC's) expert analysis recommends to give diuretics along with hydralazine and nitrates for afterload reduction. As the cardiac output increases by up to 30% in the first stage of labor and by up to 80% in the immediate postpartum period [13-14]. Systolic and diastolic blood pressure could increase with each uterine contraction. Alterations in maternal hemodynamics could change dramatically in the first 24 hours postpartum [13]. Shifts in maternal hemodynamics peak within 24-72 hours after delivery, marking the timing for the greatest risk of heart failure [13-14]. Fortunately, our patient had an uneventful delivery and was discharged home.

The management of cardiovascular disease during pregnancy is guided by risk stratification based on the Working Group on Pregnancy and Contraception classification [14]. The modified World Health Organization (WHO) classification of pregnancy risk for valvular heart disease (VHD) and some conditions associated with valve disease are specified in the European Society of Cardiology guidelines for the management of cardiovascular disease during pregnancy [15]. Seemingly, more cases of QAV associated with pregnancy are waiting to be detected to understand the natural history and, therefore, the management. Detection of the QAV during pregnancy opens a new set of concerns, including but not limited to how, and if at all, QAV needs to be managed differently in this specific patient population with dynamic hemodynamics, starting right from the time of detection to the postpartum period [12-15].

## Conclusions

QAV is a rare congenital anomaly and a unique finding in pregnancy. Due to its rarity in clinical practice, we have limited awareness and understanding for formulating a clear recommendation. Current guidelines provide recommendations only for the management of aortic regurgitation, which is the most frequent anomaly associated with QAV. However, the management of isolated QAV in pregnancy and its implication remain unknown.
